# Synergistic Responses of Forage Pea in the Germination Stage to Saline–Alkali and Drought Stress at Phenotypic, Physiological, and Non-Targeted Metabolomic Levels

**DOI:** 10.3390/biology15020131

**Published:** 2026-01-12

**Authors:** Taoxia Liu, Xiaojian Pu, Yuanyuan Zhao, Chengti Xu, Yunjie Fu

**Affiliations:** 1 College of Agriculture and Animal Husbandry, Qinghai University, Xining 810016, China; 17726933508@163.com (T.L.); puxj@qhu.edu.cn (X.P.); 2 Key Laboratory of Northwest Cultivated Land Conservation and Marginal Land Improvement Enterprises, Ministry of Agriculture and Rural Affairs, Delingha 817000, China; fszyjr@126.com; 3 Qinghai Bengsheng Grass Industry Co., Ltd., Delingha 817000, China

**Keywords:** forage pea, saline-alkali stress, drought stress, physiological and biochemical indicators, non-targeted metabolomics

## Abstract

(1) Background: This study used Qingjian No. 1 forage pea (*Pisum sativum* L.) as a plant material to study its metabolic mechanisms in response to different stresses, given that saline–alkali stress and drought stress often occur simultaneously in natural environments and severely affect the growth and yield of forage pea, while the regulatory network underlying the adaptation of forage pea to combined stress remains poorly elucidated. (2) Methods: The metabolic mechanisms of forage pea in response to different stresses were elucidated by integrating phenotypic, physiological, and metabolomic analyses. (3) Results: The results show that compared to the control, all stress treatments significantly inhibited seed germination and seedling growth, with the combined saline–alkali and drought stress exhibiting the strongest inhibitory effect. In terms of physiological and biochemical responses, peroxidase (POD) activity increased with the complexity of the stress, with the highest POD activity observed under combined saline–alkali and drought stress, showing a 61.71% increase compared to the control (*p* < 0.05). Non-targeted metabolomic analysis revealed that isoflavone biosynthesis, nucleotide metabolism, and cutin–suberin–wax biosynthesis are the core responsive pathways. Correlation analysis revealed that isocorydine and phosphatidylinositol phosphate showed strong positive correlations with the vigor index, main root length, and superoxide dismutase (SOD) activity, and glycerophospholipid metabolites were positively correlated with ferric ion-reducing antioxidant capacity. (4) This study deepens understanding of the stress resistance mechanisms in forage peas and provides a theoretical basis for stress-resistant forage pea breeding.

## Simple Summary

In natural environments, saline–alkali stress and drought stress often occur concurrently, which severely impairs the growth and yield of “Qingjian No. 1” forage pea (*Pisum sativum* L.). Using this forage pea cultivar as the experimental material, this study integrated phenotypic, physiological, and non-targeted metabolomic analyses to investigate the metabolic patterns of forage pea in response to different stresses. The results showed that all stress treatments significantly inhibited seed germination and seedling growth, with combined saline–alkali and drought stress exerting the strongest inhibitory effect. Under this combined stress, the peroxidase (POD) activity of forage pea was 61.71% higher than that of the control group (*p* < 0.05). Meanwhile, three metabolic pathways, including isoflavone biosynthesis, were identified as the core responsive pathways, and some differential metabolites were closely correlated with stress resistance-related indicators. This study enhances the understanding of stress resistance mechanisms in forage pea and holds practical value for ensuring the yield of forage pea crops and stabilizing agricultural production.

## 1. Introduction

Forage pea (*Pisum sativum* L.), as an annual herbaceous legume, holds an irreplaceable position in the supply of livestock feed and the ecological restoration of degraded grasslands on the Qinghai–Tibet Plateau, owing to its short growth cycle (typically 60–90 days), high biomass (fresh grass yield can reach 30–45 t/ha), and excellent nutritional quality (protein content of 20–25%, dietary fiber of 15–20%, and rich in flavonoid antioxidants) [1]. Seed germination, as the initial critical stage of a plant’s life cycle, directly determines the efficiency of population establishment and subsequent growth potential. This stage is particularly sensitive to abiotic stresses such as salinity and drought—even mild stress can lead to a reduction in germination rate by over 30%. In typical saline–alkali and drought-prone areas such as the Qaidam Basin in Qinghai, China, soil salinity reaches 150–200 mmol·L^−1^, with annual precipitation less than 200 mm. The dual stress factors lead to a germination rate of forage peas generally below 60%, severely constraining the sustainable development of animal husbandry [2].

In the study on the physiological regulatory mechanisms of leguminous plants in response to saline–alkali and drought stresses during germination, research on alfalfa (*Medicago sativa* L.) revealed that under the combined stress of 150 mmol·L^−1^ NaCl and 10% PEG-6000, the content of soluble sugars (such as sucrose and trehalose) in the seed hypocotyl increased by 1.8 times, showing a significant positive correlation with germination rate (r = 0.71, *p* < 0.05), which confirms the synergistic role of the multiple osmotic regulation system in response to combined stress [3,4]. In the regulation of the antioxidant defense system, different leguminous crops exhibit species-specific response characteristics. When alfalfa encounters combined saline–alkali and drought stress during the germination period, the activity of peroxidase (POD) increases by 68.4% compared to the control, and the promoter region of its encoding gene MsPOD2 contains drought-responsive elements (DREs) and salt-responsive elements (AREs), which can be activated by stress signals [5]. At the molecular level, the transcription factor-mediated stress response network is gradually becoming clear. In soybeans, the NAC transcription factor GmNAC81 shows 4.5-fold upregulation in expression under combined stress. By binding to the promoter regions of downstream genes (such as GmSOD and GmLEA), it activates pathways related to antioxidation and osmotic regulation [6]. The WRKY transcription factor PsWRKY70 in pea (Pisum sativum L.) promotes the accumulation of genistein (with a 2.1-fold increase in content) by regulating the expression of the key isoflavone synthesis gene PsCHS, thereby enhancing antioxidant capacity and ion chelation ability during seed germination [7].

Research on the responses of forage pea to saline–alkali and drought stress has preliminarily revealed its fundamental adaptive characteristics, indicating that the accumulation of osmoregulatory substances is one of the key mechanisms of pea’s salt tolerance [8]. In terms of drought stress, studies simulating a drought treatment at 50% field capacity have confirmed that peas possess a strong drought-compensation ability (evidenced by an 82.4% plant height recovery rate after 7 days of rehydration), providing a theoretical basis for water-saving cultivation [9]. However, existing research exhibits significant limitations: Firstly, the majority of studies focus on single stress factors, with insufficient analysis of the synergistic mechanisms involving “ion toxicity-osmotic stress-oxidative damage” under combined saline–alkali and drought stresses. Secondly, most research remains at the phenotypic and physiological levels, lacking support from omics technologies such as metabolomics, making it difficult to reveal the molecular regulatory networks of stress responses. Thirdly, the research materials predominantly consist of introduced varieties, with limited targeted studies on local varieties from the Qinghai–Tibet Plateau (such as those tolerant to high-altitude and barren conditions). Therefore, this study selects “Qingjian No. 1”, a local superior variety adapted to the Qinghai–Tibet Plateau, to address the regional applicability gap of previous research. Although studies have found that stress-tolerant pea genotypes could enhance composite stress tolerance by activating the catalase (CAT) and glutathione reductase (GR) systems, they did not address the regulatory mechanisms at the metabolic level, nor did they clarify the associations between key regulatory metabolites and phenotypes or physiological indicators [10].

The Qaidam Basin in Qinghai Province, China, is a typical region of combined saline–alkali and drought stress. In this environment, the germination of forage pea seeds faces a triple dilemma of “high salt inhibiting water absorption-drought exacerbating dehydration-ion toxicity damaging membrane structures”. Qingjian No. 1 is a superior forage pea variety jointly selected by the Qinghai Academy of Animal and Veterinary Sciences and the Northwest Institute of Plateau Biology, Chinese Academy of Sciences. Across years of regional trials, it has demonstrated good stress-resistance potential. However, the performance of this variety in severe composite stress regions such as the Qaidam Basin remains unsatisfactory, and the regulatory mechanisms underlying its response to combined saline–alkali and drought stress have not yet been clarified, which limits the promotion and application of the variety as well as its stress resistance improvement.

Based on the aforementioned research gaps and production demands, this study utilized Qingjian No. 1 forage pea as the material. By simulating the saline–alkali, drought, and combined saline–alkali and drought stress environments of the Qaidam Basin, it integrated phenotypic observations, physiological and biochemical assays, and non-targeted metabolomics. This study systematically measured the germination indicators, seedling growth indicators, physiological and biochemical indicators, and differential metabolites of Qingjian No. 1 pea seeds. It quantified the effects of combined saline–alkali and drought stress on the phenotypic and physiological indicators of Qingjian No. 1 during the germination period, identified the core differential metabolites regulating the response to combined saline–alkali and drought stress, and revealed the synergistic regulatory mechanisms of pathways such as isoflavone biosynthesis, providing biomarkers for stress-resistant breeding.

In response to the aforementioned research gap, this study proposes the following hypothesis: forage pea alleviates damage by regulating specific metabolic pathways under combined stress. Based on this, treatments including control, drought, saline–alkali, and combined stress were set up to analyze response mechanisms from the perspectives of phenotype, physiology, and metabolomics, providing technical support for the stress-resistant cultivation of regional forage crops.

## 2. Materials and Methods

### 2.1. Test Materials

The test material was Qingjian No. 1 forage pea seeds, with a thousand-seed weight of 153.15 g, provided by Qinghai Xinrui Agriculture and Animal Husbandry Development Co., Ltd. (11th Floor, Building B, No. 198 Xinping Avenue, Ping’an District, Haidong City, Qinghai Province, China). After manual screening, seeds that were plump, free from pest and disease damage, and uniform in size were selected and stored in a sealed container at 4 °C to maintain seed viability. The experiment was conducted in June 2025 at the Plant Physiology Laboratory of Qinghai Academy of Animal Science and Veterinary Medicine, with the laboratory environment temperature controlled at 23–25 °C and relative humidity maintained at 45–50%.

### 2.2. Experimental Design

#### 2.2.1. Preparation of Stress Treatment Solution

Drought Stress Solution (D): polyethylene glycol-6000 (PEG-6000, analytical grade, Sigma-Aldrich, St. Louis, MO, USA) was used to simulate drought stress. A 15% PEG-6000 solution was prepared based on the mass-to-volume ratio (the osmotic potential of the 15% PEG-6000 solution is −0.52 MPa, simulating a mild drought environment in the Qaidam Basin. Although PEG simulates osmotic stress, which differs from field drought in terms of soil structure changes and water conduction differences, this method has been widely used in studies of the drought stress response of leguminous plants. It can effectively eliminate interference from irrelevant variables, such as soil texture, and focus on the direct effects of drought stress on seeds. Its results have reference value for pea cultivation under actual field drought stress. A total of 15 g of PEG-6000 powder was weighed, slowly added to distilled water while stirring, and the volume was brought to 100 mL. It was stored at 4 °C to prevent PEG precipitation and allowed to return to room temperature before use.

Salt–alkali stress solution (SA): A mixed salt solution was used to simulate salt–alkali stress, with the total concentration set at 150 mmol·L^−1^. The molar ratio of each component in the mixed salt was set as NaHCO_3_:NaCl:Na_2_SO_4_:Na_2_CO_3_ = 9:3:3:1 [11] (the stress intensity at this concentration is consistent with the actual moderate stress conditions in local fields and has been verified by preliminary experiments: the germination rate of pea seeds under this intensity ranges from 40% to 60%, which can effectively distinguish the differential responses of different stress treatments, avoiding complete failure of seed germination due to excessively strong stress or no significant differences due to excessively weak stress). Each salt reagent (analytical grade, Sinopharm Chemical Reagent Co., Ltd., Shanghai, China) was accurately weighed separately, dissolved in distilled water, and diluted to 100 mL. The solution was stirred for 30 min using a magnetic stirrer (Model 85-2, Xicheng Xinrui Instrument Factory, Jintan District, Changzhou, Jiangsu 213200, China) until completely dissolved, then let stand for use.

Salt–alkali drought composite stress solution (D + SA): Mix a 15% PEG-6000 solution with a 150 mmol·L^−1^ salt–alkali mixed solution in a 1:1 (*v*/*v*) ratio. After thorough mixing, the solution is ready for use. This solution simulates both the osmotic stress induced by drought and the ionic environment of salt–alkali stress.

Control (CK): Use distilled water as the control to ensure that the only difference with each stress treatment solution is the stress factor, while other conditions remain consistent.

#### 2.2.2. Seed Treatment and Cultivation

The screened Qingjian No. 1 pea seeds were selected, and surface disinfection was performed using 75% ethanol (analytical grade, Sinopharm Chemical Reagent Co., Ltd., Shanghai, China): The seeds were placed in a sterile Erlenmeyer flask, with 75% ethanol solution added to submerge the seeds, and oscillated for 3 min on a shaker (model THZ-300, Taicang Laboratory Equipment Factory, Taicang, Jiangsu, China) at a speed of 120 r/min, with continuous stirring to ensure uniform disinfection. The seeds were then rinsed with sterile distilled water 5–6 times until the pH of the rinse solution became neutral to remove residual ethanol. The sterilized seeds were transferred to a Petri dish lined with sterile filter paper, and after absorbing surface moisture, they were soaked in sterile water at room temperature for 6 h, with stirring every 2 h to ensure uniform water absorption by the seeds.

Prepare 90 mm sterile Petri dishes, lining each dish with two layers of quantitative filter paper (90 mm in diameter, Hangzhou Special Paper Industry Co., Ltd., Hangzhou, Zhejiang, China) as a germination bed. Moisten the filter paper with sterile distilled water and drain off the excess. Evenly arrange the soaked seeds in the dishes, with 30 seeds per dish, maintaining consistent spacing (approximately 1 cm) to avoid mutual obstruction affecting germination. Add 10 mL of the corresponding treatment solution (saline–alkali stress solution, drought stress solution, saline–alkali and drought combined stress solution, distilled water) to each Petri dish, with three biological replicates for each treatment.

Place the culture dishes in an intelligent light incubator (GXZ type, Ningbo Jiangnan Instrument Factory, Haishu District, Ningbo, Zhejiang, China) for cultivation, with the culture conditions set as follows: temperature 25 °C, light intensity 3000 lx, photoperiod 12 h light/12 h dark, and relative humidity 60–65%. Weigh the dishes daily at 9:00 AM, and replenish the corresponding treatment solution based on weight loss to ensure a constant solution concentration in each dish and maintain a stable stress intensity. Simultaneously, observe and record seed germination status, and promptly remove contaminated samples.

### 2.3. Measurement Indicators and Methods

#### 2.3.1. Germination Index Measurement

Starting from the moment the seeds are placed in the incubator, the number of germinated seeds (with the criterion for germination being the radicle breaking through the seed coat by ≥2 mm) is recorded daily at 5:00 PM for a continuous period of 8 days. After 8 days of cultivation, the following germination indices are calculated in accordance with the “Rules for Agricultural Seed Testing” (GB/T 3543.4-1995):

Germination potential (%) = Number of seeds germinated in the first 4 days/Total number of tested seeds × 100%;

Germination rate (%) = Total number of germinated seeds within 8 days/Total number of tested seeds × 100%;

Germination Index (GI) = ∑(Gt/Dt), where Gt is the number of germinated seeds on day t, and Dt is the corresponding germination day;

Vigor Index (VI) = Germination Index × (Main Root Length + Shoot Length), where main root length and shoot length are the average lengths of seedlings measured on the 8th day.

#### 2.3.2. Growth Index Measurement

After cultivating for 8 days, randomly select 10 seedlings with consistent growth from each Petri dish, and measure the growth indicators using the following methods:

Primary root length: measure the straight-line distance from the base of the seed to the tip of the primary root using a vernier caliper (accuracy 0.01 cm, Shanghai Measuring & Cutting Tool Works, Songjiang District, Shanghai, China), precise to 0.1 cm.

Bud length: measure the straight-line distance from the base of the seed to the tip of the bud using a vernier caliper (accuracy 0.01 cm, Shanghai Measuring & Cutting Tool Works), accurate to 0.1 cm.

Number of lateral roots: count the total number of lateral roots on the main root of each seedling, with the counting object being the lateral roots on the main root of each seedling that are ≥0.5 cm in length.

Fresh weight: the selected seedlings were dried with absorbent filter paper to remove surface moisture, then weighed on an electronic balance (MS105DU model, Mettler-Toledo Instruments Co., Ltd., Xuhui District, Shanghai, China) with an accuracy of 0.01 g. After measurement, the seedlings were immediately placed in liquid nitrogen for preservation and used for subsequent physiological index and metabolomic analysis.

#### 2.3.3. Determination of Physiological and Biochemical Indicators

Malondialdehyde (MDA) content: determined using the thiobarbituric acid (TBA) colorimetric method. Weigh 0.5 g of liquid nitrogen-preserved seedling sample, add 5 mL of 5% trichloroacetic acid (TCA, analytical grade, Sinopharm Chemical Reagent Co., Ltd., Huangpu District, Shanghai, China), and homogenize using a tissue homogenizer (Model MM 400, Retsch, Verder Retsch (Shanghai) Trading Co., Ltd., Room 302, Tower 1, No. 289 Bisheng Road, Zhangjiang Hi-Tech Park, Pudong New Area, Shanghai, China) in an ice bath (30 Hz, 2 min). Centrifuge the homogenate at 4 °C and 10,000× *g* for 15 min, take 2 mL of the supernatant, add 2 mL of 0.67% TBA solution, mix well, and heat in a boiling water bath for 30 min. After cooling to room temperature, measure the absorbance at 532 nm and 600 nm (Model UV-2600, Shimadzu Instruments Co., Ltd., Shimadzu Corporation, 1 Nishinokyo Kuwabara-cho, Nakagyo-ku, Kyoto, Japan). The MDA content is calculated using the following formula: MDA (μmol·g^−1^ FW) = [6.45 × (A532 − A600) − 0.56 × A450] × Vt/(Vs × m), where Vt is the total volume of the extraction solution, vs. is the volume of the extraction solution used for measurement, and m is the fresh weight of the sample.

Superoxide dismutase (SOD) activity: determined using the nitroblue tetrazolium (NBT) photoreduction method. Weigh 0.5 g of seedling sample, add 5 mL of 50 mmol·L^−1^ phosphate buffer (pH 7.8, containing 1% polyvinylpyrrolidone), and grind into a homogenate in an ice bath; centrifuge at 4 °C and 10,000× *g* for 20 min, and the supernatant is the crude enzyme extract. The reaction system consists of 50 mmol·L^−1^ phosphate buffer, 13 mmol·L^−1^ methionine, 75 μmol·L^−1^ NBT, 10 μmol·L^−1^ EDTA-Na_2_, 2 μmol·L^−1^ riboflavin, and crude enzyme extract. After thorough mixing, the reaction is carried out under 4000 lx light illumination for 20 min, and the absorbance at 560 nm wavelength is measured. The amount of enzyme required to inhibit 50% of NBT photoreduction is defined as one enzyme activity unit (U). The SOD activity (U·g^−1^ FW) is then calculated.

Peroxidase (POD) activity: determined using the guaiacol method. The preparation of the crude enzyme extract was the same as that for SOD determination. The reaction system consisted of 50 mmol·L^−1^ phosphate buffer (pH 6.0), 20 mmol·L^−1^ guaiacol, 40 mmol·L^−1^ H_2_O_2_, and the crude enzyme extract. After incubation in a 37 °C water bath for 5 min, the change in absorbance at 470 nm was measured (an increase in absorbance of 0.01 per minute was defined as one unit of enzyme activity), and the POD activity (U·g^−1^·min^−1^) was calculated.

Catalase (CAT) activity: determined using the hydrogen peroxide method. The preparation of the crude enzyme extract was the same as that for the SOD assay. The reaction system included 50 mmol·L^−1^ phosphate buffer (pH 7.0), 20 mmol·L^−1^ H_2_O_2_, and the crude enzyme extract. The change in absorbance at 240 nm wavelength was measured (the molar extinction coefficient of H_2_O_2_ is 39.4 mmol^−1^·cm^−1^), and the CAT activity (U·g^−1^·min^−1^) was calculated, where 1 U is defined as the amount of enzyme required to decompose 1 μmol H_2_O_2_ per minute.

Hydrogen peroxide (H_2_O_2_) content: determined according to the instructions of the H_2_O_2_ assay kit from Suzhou Keming Biotechnology Co., Ltd., Unit 101, Building A2, BioBAY, No. 218 Xinghu Street, Suzhou Industrial Park, Suzhou, Jiangsu, China Weigh 0.5 g of seedling sample, add 5 mL of pre-chilled acetone, and homogenize by grinding in an ice bath; centrifuge at 4 °C and 8000× *g* for 10 min, take the supernatant, react according to the kit procedure, measure the absorbance at 415 nm wavelength, and calculate the H_2_O_2_ content (nmol·g^−1^ FW) based on the standard curve.

All physiological and biochemical indicators were measured with three technical replicates, and the average values were used for statistical analysis.

### 2.4. Sample Collection and Extraction Process for Non-Targeted Metabolomics Analysis During Seed Germination

#### 2.4.1. Sample Collection and Preservation

On the 8th day of the germination test, approximately 1 g of seedling samples (including roots and shoots) with consistent growth was randomly selected from each replicate of each treatment and quickly placed in liquid nitrogen for rapid freezing for 15 min to terminate metabolic reactions and prevent metabolite degradation. The frozen samples were then transferred to a −80 °C ultra-low temperature freezer (model DW-86L388, Haier Group Corporation, Haier Industrial Park, No. 1 Haier Road, Laoshan District, Qingdao, Shandong, China) for storage, ready for subsequent metabolomic analysis.

#### 2.4.2. Sample Pretreatment

Transfer the samples stored at −80 °C to a freeze dryer (Model Scientz-100F, Ningbo Scientz Biotechnology Co., Ltd., No. 65 Mujin Road, Ningbo National Hi-Tech Park, Yinzhou District, Ningbo, Zhejiang, China) and vacuum freeze-dry them at −50 °C and 0.01 mbar for 63 h until the samples are completely dehydrated. Grind the dried samples in a grinder (Model MM 400, RETSCH GmbH, Retsch-Allee 1-5, 42781 Haan, Nordrhein-Westfalen, Germany) at 30 Hz for 1.5 min to produce a homogeneous powder, and sieve through a 100-mesh screen to remove impurities.

Accurately weigh 30 mg of sample powder using an electronic balance (Model MS105DU, Mettler-Toledo Instrument Co., Ltd., No. 589 Guiping Road, Xuhui District, Shanghai, China), and transfer it to a 2 mL centrifuge tube (Corning Life Sciences Co., Ltd., Shanghai, China). Add 1500 μL of a pre-chilled −20 °C 70% methanol aqueous internal standard extraction solution (internal standard: 250 μg·mL^−1^ L-2-chlorophenylalanine, purchased from Sigma-Aldrich Co., St. Louis, MO, USA). If the sample powder is less than 30 mg, adjust the volume of the extraction solution according to the ratio of “1500 μL of extraction agent per 30 mg of sample”. After mixing the sample with the extraction solution, vortex for 30 s, then vortex once every 30 min (30 s each time), for a total of 6 times, ensuring thorough dissolution of metabolites; subsequently centrifuge at 4 °C and 12,000× *g* for 3 min, aspirate 1000 μL of supernatant, filter through a 0.22 μm organic phase microporous filter membrane (Shanghai Anpel Experimental Technology Co., Ltd., No. 59 Yezhang Road, Yexie Town, Songjiang District, Shanghai, China), collect the filtrate into vials, and use for ultra-performance liquid chromatography–tandem mass spectrometry (UPLC-MS/MS, Orbitrap platform in Thermo Fisher Scientific Inc., Waltham, MA, USA) non-targeted metabolomic analysis.

#### 2.4.3. UPLC-MS/MS Analysis Conditions

All samples were acquired by the LC-MS system following machine orders. The analytical conditions were as follows: UPLC: column, Waters ACQUITY UPLC HSS (Waters, Shanghai, China) T3 1.8 µm, 2.1 mm ×100 mm; column temperature, 40 °C; flow rate, 0.40 mL/min; injection volume, 4 µL; solvent system, water (0.1% formic acid): acetonitrile (0.1% formic acid). Sample measurements were performed with a gradient program that employed the starting conditions of 95% A, 5% B. Within 5 min, a linear gradient to 35% A, 65% B was programmed. Within 1 min, a linear gradient to 1% A, 99% B was programmed and maintained for 1.5 min. Subsequently, a composition of 95% A, 5.0% B was adjusted within 0.1 min and maintained for 2.4 min.

All the methods alternated between full-scan MS and data-dependent MSn scans using dynamic exclusion. MS analyses were carried out using electrospray ionization in the positive ion mode and negative ion mode using full-scan analysis over m/z 84–1250 at a 35,000 resolution. Additional MS settings include the ion spray voltage, 3.5 KV or 3.2 KV in positive or negative modes, respectively; sheath gas (Arb), 30; Aux gas, 5; ion transfer tube temperature, 320 °C; vaporizer temperature, 300 °C; collision energy, 30, 40, 50 V; Signal Intensity Threshold, (10 × 10^−5^) cps; top N vs. top speed, 10; exclusion duration, 3 s.

### 2.5. Data Processing

The experimental data were organized and preliminarily calculated using Excel 2019 software for non-targeted metabolomic data preprocessing: peak extraction, alignment, and integration were performed using Progenesis QI software (v2.3, Nonlinear Dynamics, Waters Corporation, Newcastle, UK). Metabolite peak areas were normalized to the internal standard (L-2-chlorophenylalanine) to correct for variations in extraction and injection volume. Batch effects were adjusted using the quantile normalization method. Metabolite identification was based on the following criteria: retention time tolerance of ± 0.2 min compared with standard substances; mass accuracy < 5 ppm, and fragment ion ratio matching with the Human Metabolome Database (HMDB) and Metlin database (match score > 80), with the results expressed as “Mean ± Standard Error (Mean ± SE)”. One-way analysis of variance (ANOVA) and *t*-tests were conducted using Origin 2024 software to analyze the significance of differences among different treatments, with the significance level set at *p* < 0.05 (significant) and *p* < 0.01 (highly significant). Principal component analysis (PCA), orthogonal partial least squares-discriminant analysis (OPLS-DA), and hierarchical cluster analysis (HCA) were performed using R language (v4.3.0), with the HCA heatmap drawn using the pheatmap package (v1.0.12). The correlation analysis between metabolites and phenotypes or physiological indicators was visualized using the corrplot package (v0.92). The screening criteria for differential metabolites included a projection (VIP) value >1 in the OPLS-DA model and a *p*-value <0.05 in the *t*-test.

## 3. Results

### 3.1. Effects of Different Stresses on Phenotypic Traits of Forage Pea During Germination

**Figure 1:**
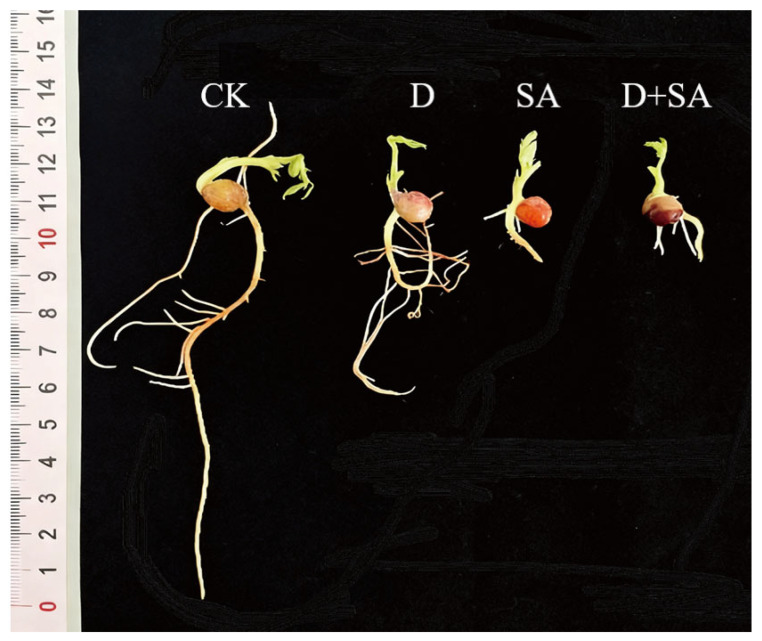
Morphological changes of roots and shoots of forage pea under different stress treatments. In the figure, CK, D, SA, and D + SA represent control, drought stress, saline–alkali stress, and combined stress, respectively.

**Figure 2:**
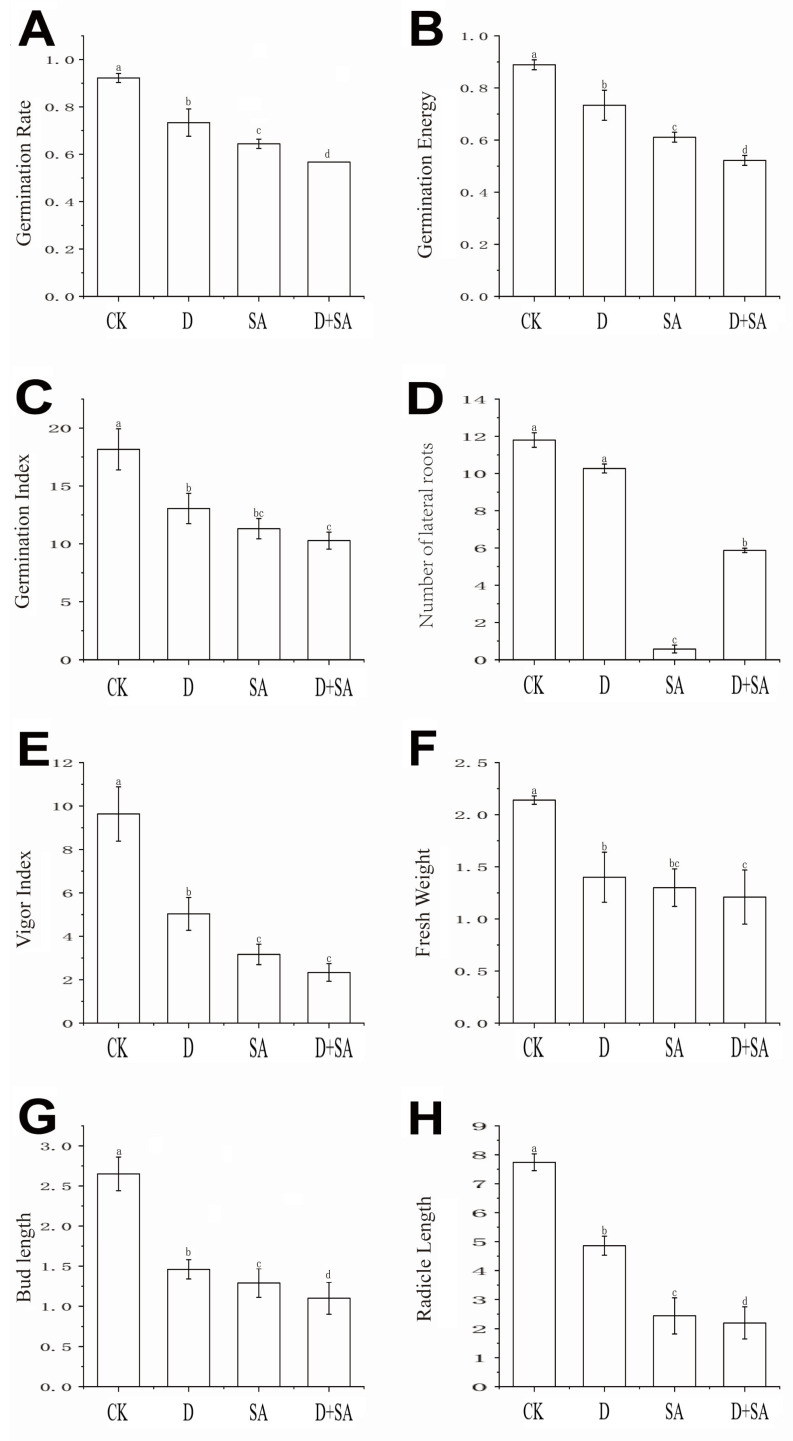
Germination and seedling growth-related indices of Qingjian No. 1. (**A**) Germination rate; (**B**) germination potential; (**C**) germination index; (**D**) number of lateral roots; (**E**) vigor index; (**F**) fresh weight; (**G**) shoot length; (**H**) radicle length. In the figure, CK, D, SA, and D + SA represent control, drought stress, saline–alkali stress, and combined stress, respectively. Data are expressed as “mean ± standard error” (n = 3). One-way ANOVA was performed on the measured values of different treatment groups, and different lowercase letters indicate significant differences between treatments at *p* < 0.05, while the same letters indicate no significant differences between groups.

Seeds of forage pea cultivar Qingjian No. 1 were subjected to salt–alkali stress, drought stress, and combined stress during the germination stage, and their phenotypic traits were analyzed (as shown in Figure 1). As indicated in Figure 2, compared with the CK group, the three stress treatments (drought, salt–alkali, and combined stress) caused significant differences (*p* < 0.05) in eight germination-related indicators, including germination rate, germination energy, germination index, radicle length, shoot length, number of lateral roots, fresh weight, and vigor index. All stress treatments significantly inhibited the germination process of pea seeds relative to the CK group. Regarding the germination rate (Figure 2A), it decreased by 18.83%, 27.77%, and 35.50% in the D (drought), SA (salt–alkali), and D + SA (combined stress) groups, respectively. Regarding germination energy (Figure 2B), the corresponding reductions were 15.53%, 27.80%, and 36.70% in the three stress groups. As shown in Figure 2C, the germination index of the CK group was 18.16 ± 1.77, which sequentially decreased to 13.05 ± 1.30, 11.31 ± 0.88, and 10.28 ± 0.74 in the D, SA, and D + SA groups. The growth of radicles and plumules was significantly affected by the stresses. The radicle length of the CK group was 7.74 ± 0.29 cm, which was shortened by 37.2% and 68.5% in the D and SA groups, respectively. The main root length in the D + SA group was 2.20 ± 1.40 cm, representing a further reduction of 8.2% compared with the SA group. The shoot length showed a similar variation trend. The number of lateral roots is an important indicator for evaluating root system development. The CK group had 11.80 ± 0.89 lateral roots, while this number drastically dropped to 0.57 ± 0.21 in the SA group and recovered to 5.87 ± 1.12 in the D + SA group. Fresh weight and vigor index decreased synchronously. The fresh weight and vigor index of the CK group were 2.14 ± 0.04 g and 9.63 ± 1.25, respectively. There was no significant difference in fresh weight between the D + SA group (1.21 ± 0.26 g) and the SA group (1.30 ± 0.18 g) (*p* > 0.05), but both were significantly lower than that of the CK group. Although the vigor index of the D + SA group (3.17 ± 0.47) was higher than that of the SA group, it was still significantly lower than that of the CK group (*p* < 0.05).

### 3.2. Effects of Different Stresses on Physiological Indicators of Forage Pea During Germination

**Figure 3:**
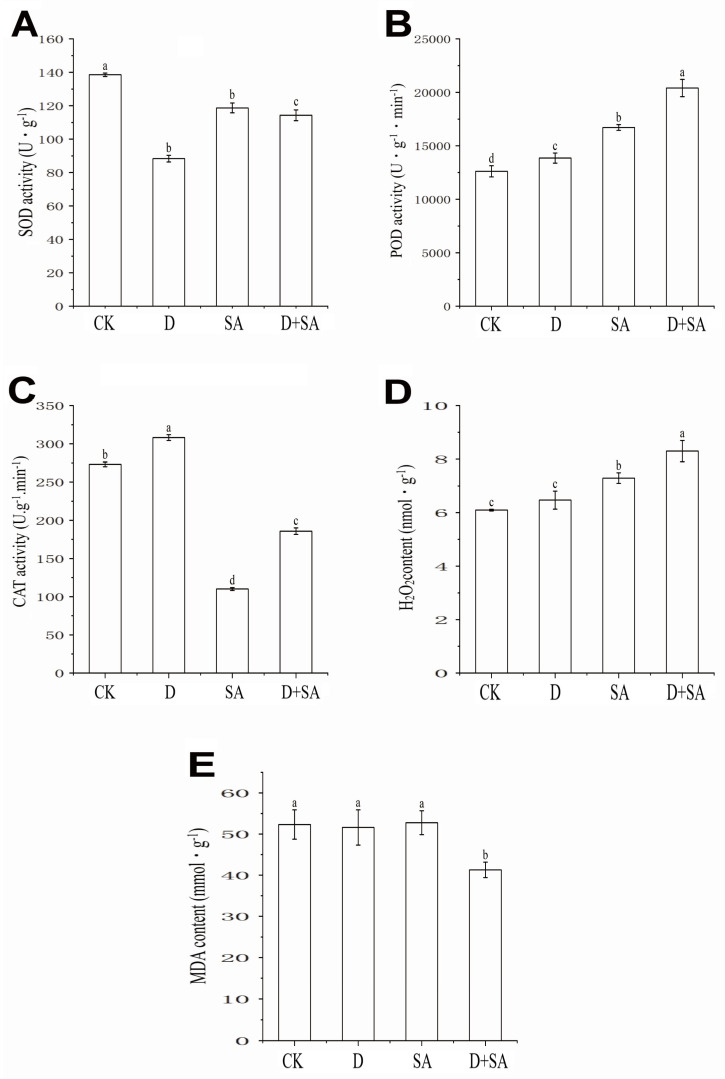
Effects of different treatments on physiological and biochemical indices of forage pea. (**A**) Superoxide dismutase (SOD) activity; (**B**) peroxidase (POD) activity; (**C**) catalase (CAT) activity; (**D**) hydrogen peroxide (H_2_O_2_) content; (**E**) malondialdehyde (MDA) content. In the figure, CK, D, SA, and D + SA represent control, drought stress, saline–alkali stress, and combined stress, respectively. Data are presented as “mean ± standard error” (n = 3). One-way analysis of variance (ANOVA) was performed on the measured values of different treatment groups. Different lowercase letters indicate significant differences between treatments at *p* < 0.05, while the same letters indicate no significant differences between groups.

As shown in Figure 3A, there were significant differences in the superoxide dismutase (SOD) activity of forage pea under different treatments. The CK treatment group exhibited the highest SOD activity, which was (139.2 ± 3.5) U·g^−1^. In contrast, the SOD activity in the D treatment group was significantly reduced (*p* < 0.05), only reaching (89.5 ± 2.8) U·g^−1^. The SOD activity in the SA treatment group showed a recovery, reaching (119.8 ± 3.2) U·g^−1^, but it was still significantly lower than that in the CK treatment group (*p* < 0.05). For the D + SA treatment group, the SOD activity was (114.3 ± 2.9) U·g^−1^, which was significantly higher than that in the D treatment group (*p* < 0.05) but significantly lower than that in the CK treatment group (*p* < 0.05).

As shown in Figure 3B, there were significant differences in the peroxidase (POD) activity of forage pea under different treatments. Taking the mean POD activity of the CK treatment group (12,618.42 U·g^−1^·min^−1^) as the control, POD activity in all treatment groups was significantly increased: the D treatment group showed an increase of 9.79%, the SA treatment group exhibited a rise of 32.50%, and the D + SA treatment group had an increase of 61.71%, which was significantly higher than that in the other single-treatment groups.

As shown in Figure 3C, there were significant differences in the catalase (CAT) activity of forage pea under different treatments. CAT activity in the CK treatment group was (275.3 ± 12.5) U·g^−1^·min^−1^. In contrast, CAT activity in the D treatment group was significantly increased (*p* < 0.05), reaching (310.5 ± 10.2) U·g^−1^·min^−1^. However, CAT activities in the SA and D + SA treatment groups were significantly decreased (*p* < 0.05), only being (110.2 ± 8.3) U·g^−1^·min^−1^ and (188.5 ± 9.6) U·g^−1^·min^−1^, respectively.

As shown in Figure 3D, there were significant differences in the hydrogen peroxide (H_2_O_2_) content of forage pea under different treatments. Taking the mean H_2_O_2_ content of the CK treatment group as 6.096 μmol·g^−1^, the values in the D, SA, and D + SA treatment groups increased by 6.14%, 16.32%, and 36.15%, respectively.

As shown in Figure 3E, there were significant differences in the malondialdehyde (MDA) content of forage pea under different treatments. No significant difference was observed in MDA content among the CK, D, and SA treatment groups (*p* > 0.05), with values of (52.8 ± 2.1) nmol·g^−1^, (51.5 ± 2.5) nmol·g^−1^, and (53.2 ± 1.8) nmol·g^−1^, respectively. In contrast, the MDA content in the D + SA treatment group was significantly reduced (*p* < 0.05), only reaching (41.6 ± 1.5) nmol·g^−1^.

### 3.3. Non-Targeted Metabolomic Analysis of Forage Pea in Response to Different Stresses During the Germination Stage

#### 3.3.1. Overall Quality Control of Samples and Principal Component Analysis

**Figure 4:**
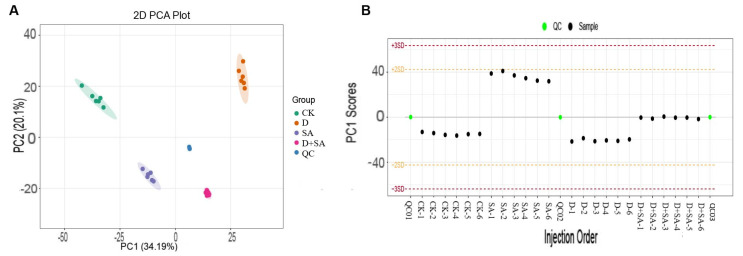
Principal component analysis (PCA) of metabolomic characteristics. (**A**) Two-dimensional PCA plot showing the metabolic separation among experimental groups (CK, SA, D, D + SA, and QC); (**B**) PCA score stability of quality control and sample injections in the sequence of injection.

Principal component analysis (PCA) revealed that the first two principal components (PC1 and PC2) accounted for 34.19% and 20.1% of the total variance, respectively (Figure 4A). The distribution of samples in the PCA plot showed distinct clustering among the groups, indicating significant metabolic heterogeneity between these groups. The established PCA model was used to monitor the QC samples (Figure 4B). All QC samples were tightly clustered near the center, and within the ±3 standard deviation range, the relative standard deviation (RSD) of the internal standard peak area across all samples was <5%, and the RSD of metabolite peaks in QC samples was <15%, indicating good stability and reproducibility of the non-targeted metabolomic analysis platform. These results collectively demonstrate that the metabolic data are reliable and suitable for subsequent differential metabolite analysis.

#### 3.3.2. Orthogonal Partial Least Squares Discriminant Analysis (OPLS-DA) and Permutation Test for Different Treatment Groups

**Figure 5:**
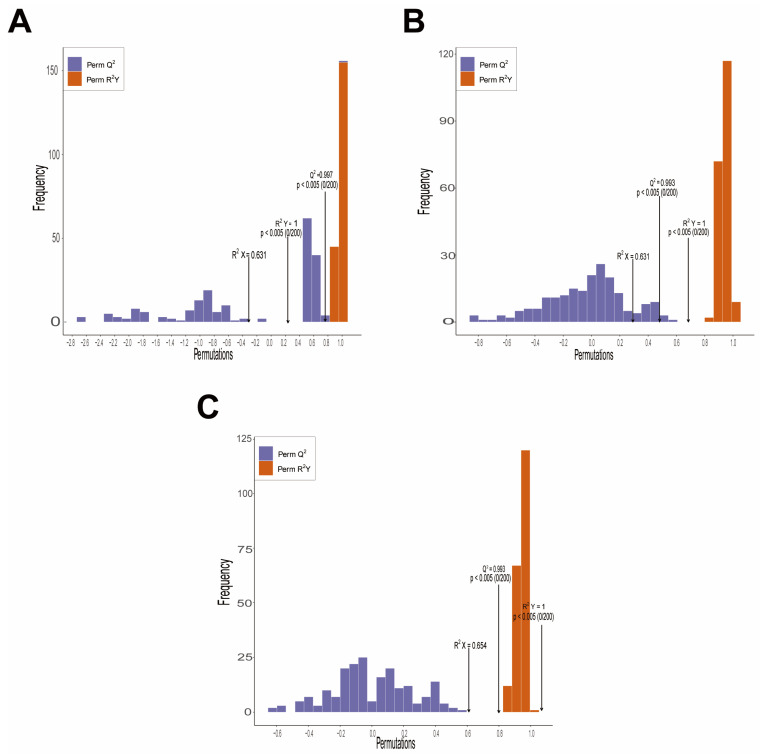
Permutation tests of Orthogonal Partial Least Squares Discriminant Analysis (OPLS-DA) models used for different experimental comparisons. (**A**) Permutation test for CK vs. D; (**B**) permutation test for CK vs. SA; (**C**) permutation test for CK vs. D + SA. All plots show distributions of permuted R^2^Y and Q^2^, with statistical significance (*p* < 0.05, n = 200 permutations) indicating valid model predictability.

Orthogonal Partial Least Squares Discriminant Analysis (OPLS-DA) further enhances the ability to distinguish between groups (Figure 5). The pairwise comparison results revealed significant metabolic differences between CK and D (R^2^X = 0.631, R^2^Y = 1.000, Q^2^ = 0.997), CK and SA (R^2^X = 0.631, R^2^Y = 1.000, Q^2^ = 0.993), and CK and D + SA (R^2^X = 0.654, R^2^Y = 1.000, Q^2^ = 0.993). A 200-permutation test confirmed the absence of overfitting in the model (*p* < 0.05). These results collectively validate the reliability and robustness of the multivariate statistical analysis.

#### 3.3.3. Differential Metabolite Screening

**Figure 6:**
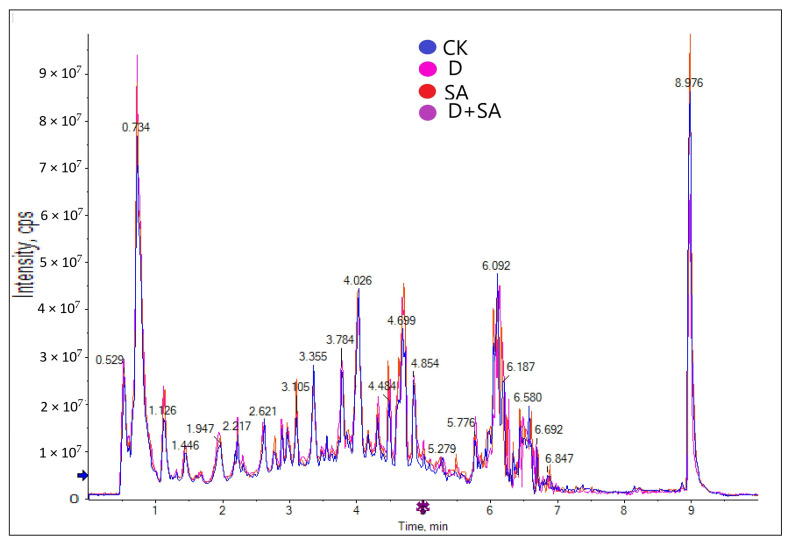
Comparison of total ion chromatograms (TICs) across sample groups.

A total of 1949 metabolites were clearly annotated from the four treatment groups, and Figure 6 shows the overlaid total ion current (TIC) chromatogram under the negative electrospray ionization mode (ESI^−^).

**Figure 7:**
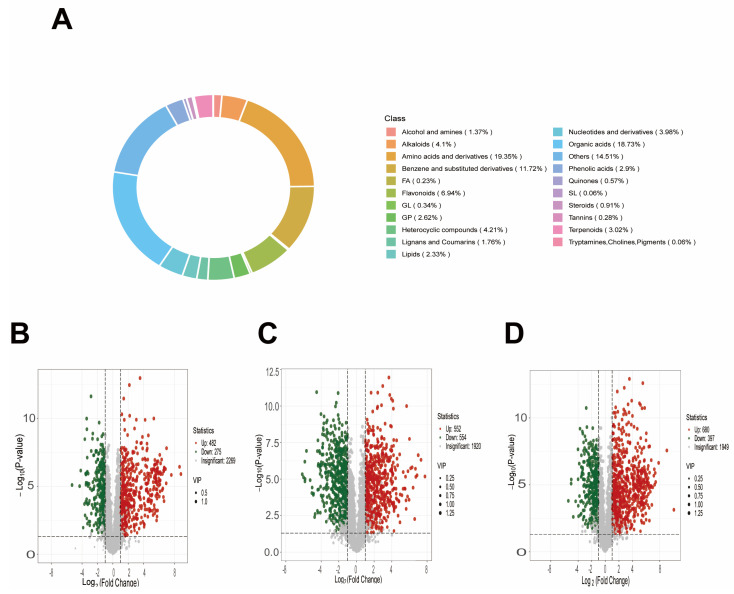
Classification of metabolites and changes in the number of differential metabolites. (**A**) Proportional distribution of metabolites; (**B**) Volcano plot of CK vs. D; (**C**) Volcano plot of CK vs. SA; (**D**) Volcano plot of CK vs. D + SA, with VIP scores and statistical thresholds annotated.

The significantly different metabolites detected in the samples are mainly categorized into organic acids (18.73%), amino acids and their derivatives and their derivatives and their derivatives (19.30%), and lipids (23.36%), which collectively account for a substantial proportion of the total metabolites. Meanwhile, other categories, including alcohols and amines, alkaloids, and phenols, also contribute to metabolic diversity (Figure 7A).

To characterize the metabolic composition and differential metabolites between groups, comprehensive metabolic profiling was conducted. The circular diagram (Figure 7A) illustrates the distribution of metabolites across different categories, with organic acids (18.73%), amino acids and their derivatives and their derivatives (19.30%), and lipids (23.36%) being the major categories. Based on the FC+VIP screening criteria, multiple sets of differential metabolites were identified: 757 between CK and D (482 upregulated, 275 downregulated), 1106 between CK and SA (552 upregulated, 554 downregulated), and 1077 between CK and D + SA (680 upregulated, 397 downregulated) (Figure 7B–D).

#### 3.3.4. Importance Analysis of Differential Metabolites

**Figure 8:**
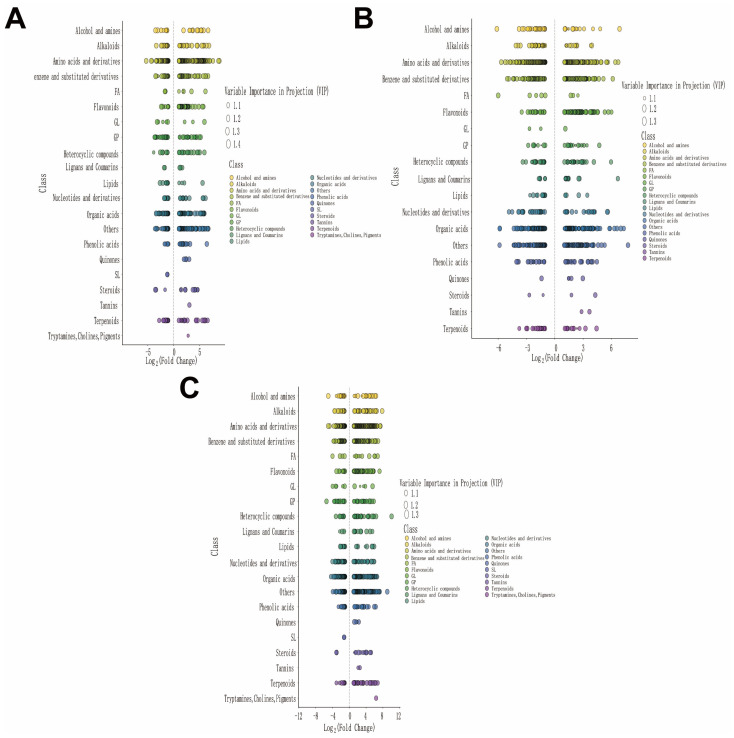
Variable Importance in Projection (VIP) plots of differentially expressed metabolites across experimental comparisons. (**A**) VIP plot for CK vs. D; (**B**) VIP plot for CK vs. SA; (**C**) VIP plot for CK vs. D + SA. Each plot displays metabolites categorized by chemical class, with VIP scores and Log_2_ (fold change) indicated.

To evaluate the differential accumulation of metabolites under different treatments and their significance, we plotted a Variable Importance in Projection (VIP) graph combined with log_2_(fold change) analysis, and the results are shown in Figure 8. Under drought stress treatment (Figure 8A), significant changes in amino acids and their derivatives and their derivatives, organic acids, and terpenoids were observed, suggesting their potential roles in mediating the response to drought stress treatment. Under salt–alkali stress treatment (Figure 8B), amino acids and their derivatives and their derivatives, nucleotides and their derivatives, and phenolic acids exhibited significant VIP values and fold changes, indicating their crucial roles in the metabolic response to salt–alkali stress treatment. Under combined saline–alkali and drought stress treatment (Figure 8C), terpenoids, amino acids and their derivatives, and organic acids were among the most significantly differentially accumulated metabolite categories, with high VIP scores, suggesting their significant contributions to the effects of combined saline–alkali and drought stress treatment.

#### 3.3.5. Differential Metabolite Cluster Analysis

**Figure 9:**
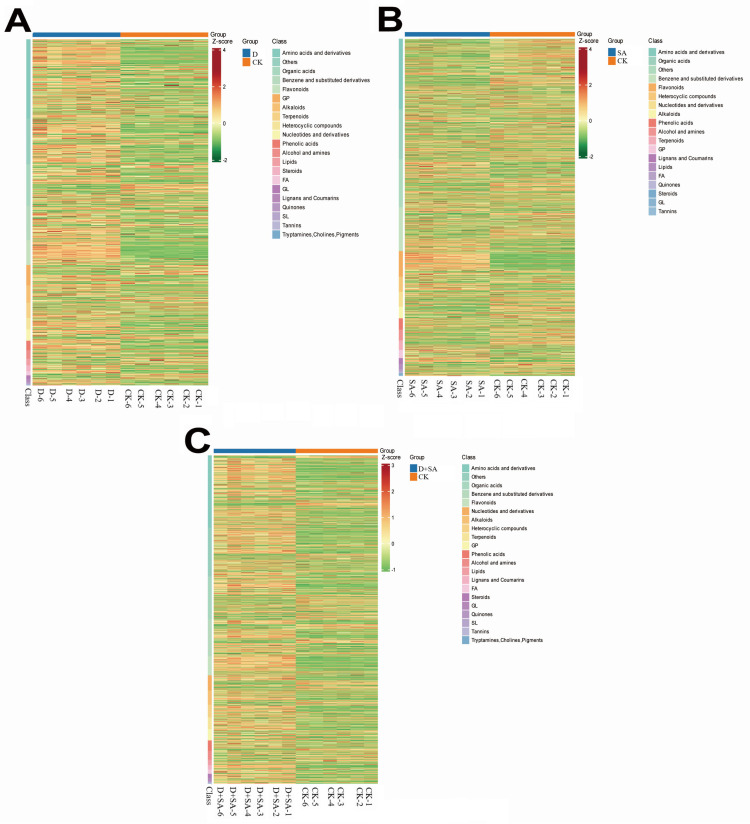
Hierarchical clustering heatmaps of metabolomic profiles across experimental comparisons. (**A**) Heatmap of CK vs. D; (**B**) heatmap of CK vs. SA; (**C**) heatmap of CK vs. D + SA. Each heatmap displays metabolite abundances (Z-score-normalized), categorized by chemical class, with experimental groups indicated.

To characterize differences in metabolic profiles under various treatments, a hierarchical clustering heatmap was constructed, and the results are shown in Figure 9. In Figure 9A, metabolites are categorized by chemical classes (such as amino acids and their derivatives and their derivatives, organic acids, and flavonoids), with their abundances standardized by Z-score. The heatmap reveals distinct clustering patterns between drought stress and the control group, with significant differences in the accumulation of amino acids and their derivatives, organic acids, and terpenoids, indicating that drought stress treatment triggered significant metabolic reprogramming. Figure 9B further illustrates the differential distribution of metabolites, with amino acids and their derivatives and their derivatives, nucleotides and their derivatives, and phenolic acids showing significant abundance changes between the two groups, suggesting that saline–alkali stress treatment induced specific metabolic responses. In Figure 9C, the heatmap demonstrates a distinct clustering of metabolites in chemical categories such as terpenoids, amino acids and their derivatives, and organic acids between the saline–alkali drought combined treatment group and the control group, with significant alterations in metabolite abundance, reflecting the synergistic effect of the saline–alkali drought combined treatment on metabolic regulation. In summary, these hierarchical clustering heatmaps identify key metabolite categories with differential accumulation under various treatments, providing a comprehensive perspective for deciphering the changes underlying metabolic responses under different experimental conditions.

#### 3.3.6. KEGG Analysis

**Figure 10:**
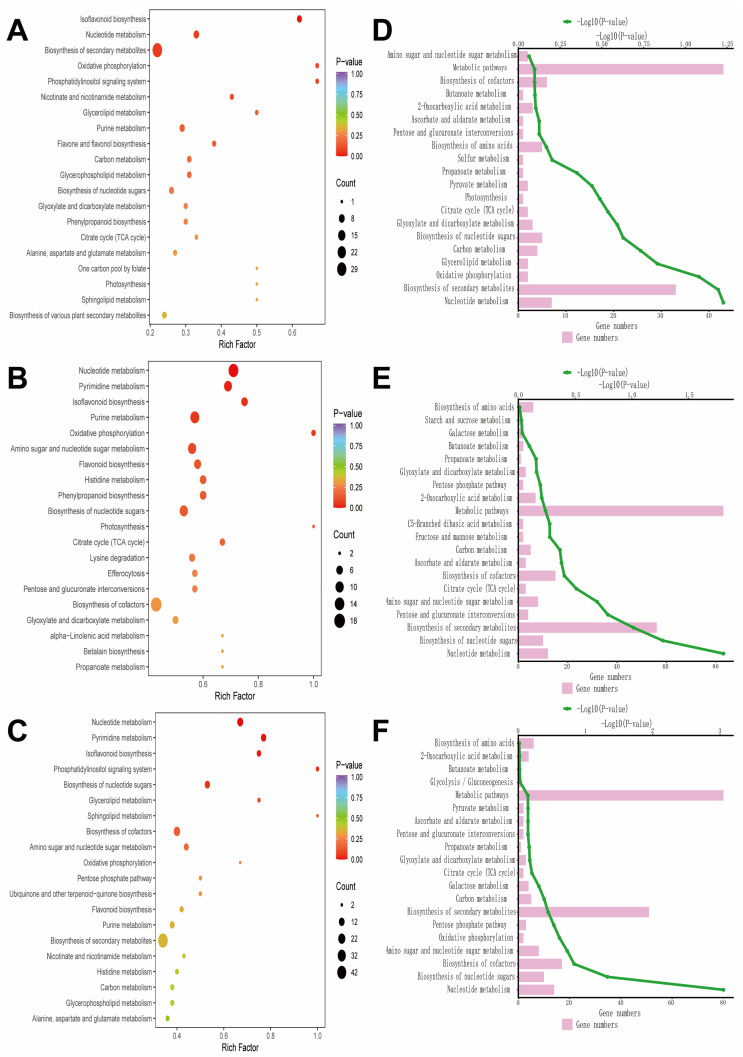
Metabolic pathway enrichment and functional classification analysis. (**A**) Bubble chart of CK vs. D; (**B**) bubble chart of CK vs. SA; (**C**) bubble chart of CK vs. D + SA, respectively, showing the enrichment factor, *p*-value, and the number of metabolites involved; (**D**) enriched pathway map of CK vs. D; (**E**) enriched pathway map of CK vs. SA; (**F**) enriched pathway map of CK vs. D + SA.

The Kyoto Encyclopedia of Genes and Genomes (KEGG) enrichment analysis revealed that metabolic differences among groups were mainly enriched in pathways related to secondary metabolite biosynthesis, nucleotide metabolism, amino acid metabolism, and energy metabolism (such as the TCA cycle and carbohydrate metabolism) (Figure 10).

The repeatedly enriched pathways include nucleotide metabolism, flavonoid biosynthesis, purine metabolism, phenylpropanoid biosynthesis, amino sugar and nucleotide sugar metabolism, tricarboxylic acid cycle, sugar metabolism, amino acid biosynthesis, pyruvate metabolism, and others. Among these, drought stress treatment primarily regulates metabolic pathways involved in substance synthesis and energy metabolism; saline–alkali stress treatment induces metabolic reprogramming, particularly notable in the biosynthesis pathways of nucleotides and secondary metabolites. The combined saline–alkali and drought stress treatment triggers a comprehensive metabolic response, with significantly enhanced enrichment in the pathways of secondary metabolites and energy metabolism compared to individual treatments.

### 3.4. Correlation Analysis

#### 3.4.1. Analysis of the Relationship Between Metabolites and Phenotypic Traits in Forage Pea Roots and Seedlings Under Different Stress Conditions

**Figure 11:**
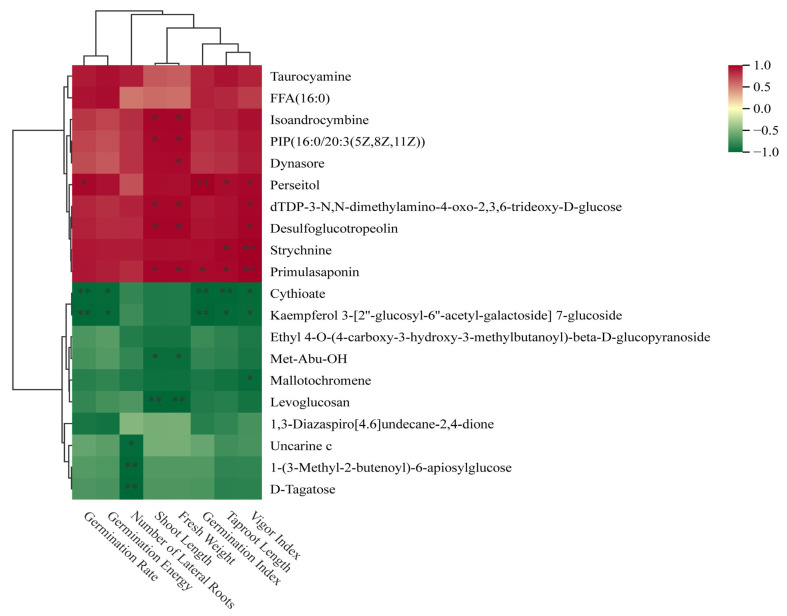
Hierarchical clustering heatmap of metabolites associated with seed germination phenotypic indices. The heatmap shows the correlation (Z-score-normalized) between differentially accumulated metabolites and phenotypic traits (vigor index, Taproot Length Index, germination rate, etc.), with red and green indicating positive and negative correlations, respectively. *: Significant correlation at *p* < 0.05; **: Significant correlation at *p* < 0.01.

The correlation analysis between twenty common differential metabolites and phenotypic traits of forage pea during the germination period revealed findings, as shown in Figure 11. Isoandrocymbine, phosphatidylinositol phosphate (PIP (16:0/20:3)), and primulasaponin exhibited strong positive correlations with growth phenotypes such as vigor index and main root length (correlation coefficient r > 0.7, *p* < 0.01). Uncarine c, D-tagatose, and levoglucosan showed significant negative correlations with the aforementioned growth traits (r < −0.6, *p* < 0.01).

#### 3.4.2. Analysis of the Relationship Between Metabolites and Physiological Indicators in Forage Pea Roots and Shoots Under Different Stress Conditions

**Figure 12:**
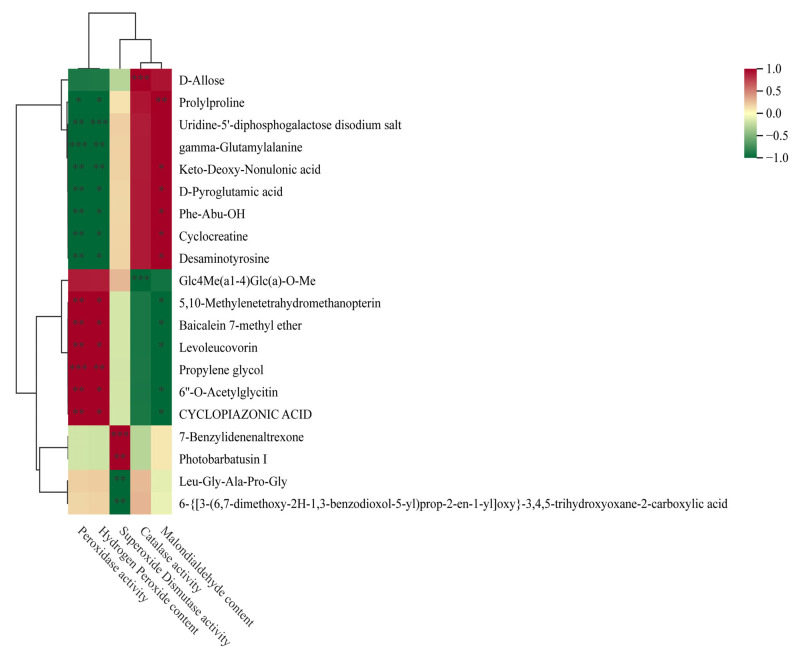
Hierarchical clustering heatmap of metabolites associated with physiological and biochemical traits. The heatmap demonstrates the correlation (Z-score-normalized) between differentially accumulated metabolites and physiological and biochemical indices (Malondialdehyde Content, Catalase Activity, Superoxide Dismutase Activity, etc.), with red and green representing positive and negative correlations, respectively. *: Significant correlation at *p* < 0.05; **: Significant correlation at *p* < 0.01; ***: Significant correlation at *p* < 0.001.

Figure 12 illustrates the correlation analysis between 20 common differential metabolites and physiological indices of forage pea during germination. Among these metabolites, multiple compounds showed distinct correlation patterns with key physiological parameters. Several metabolites (e.g., Prolyproline, D-Allose) exhibited significant positive correlations with superoxide dismutase (SOD) activity (|r| > 0.7, *p* < 0.01), which was also consistent with their positive associations with growth phenotypes. In contrast, metabolites including Desaminotyrosine and Cyclocreaine displayed an opposite regulatory pattern: they showed strong negative correlations with SOD activity (r < −0.6, *p* < 0.01) while being significantly positively correlated with malondialdehyde (MDA) content and hydrogen peroxide (H_2_O_2_) content (|r| > 0.6, *p* < 0.01). Additionally, compounds such as gamma-Glutamylalanine and Phe-Abu-OH showed moderate correlations with catalase (CAT) activity (0.5 < |r| < 0.7, *p* < 0.05), indicating their potential role in mediating the antioxidant system of germinating forage pea.

## 4. Discussion

Against the backdrop of combined saline–alkali and drought stress in the Qaidam Basin, this study systematically explored the response patterns of the local superior variety “Qingjian No. 1” forage pea during germination using an integrated approach of phenotypic observation, physiological assays, and untargeted metabolomics. For the first time, we revealed its adaptive mechanism involving “phenotypic plasticity-physiological defense-metabolic reprogramming” synergistic regulation, providing theoretical support and practical targets for stress-resistant breeding and cultivation of leguminous forages in high-altitude saline–alkali and arid regions.

Phenotypic trait analysis indicated that the inhibitory effect of combined saline–alkali and drought stress on seed germination and seedling growth of “Qingjian No. 1” was significantly stronger than that of single stress, exhibiting the characteristic of “stress factor superposition-damage effect amplification”; under combined stress, the germination rate decreased compared to both drought and saline–alkali stress, while the germination index was lower than that of the control, which is consistent with the “synergistic inhibitory effect of combined stress” reported by Chen et al. [12] in mung beans, with the core mechanism being the triple overlay of “osmotic stress-ion toxicity-oxidative damage”: drought-induced cellular dehydration exacerbates the passive absorption of Na^+^ and CO_3_^2−^ [13], increasing the permeability of root cell plasma membrane and inducing excessive production of reactive oxygen species (ROS), and correspondingly, the hydrogen peroxide (H_2_O_2_) content under combined stress was significantly higher than that under single drought stress and saline–alkali stress, which is highly consistent with the observation in soybean that “ROS accumulation increases with the complexity of stress” [14].

“Qingjian No. 1” exhibits a unique phenotypic adaptive trade-off under combined stress: the primary root length is shorter than that of the control, while the number of lateral roots is significantly recovered compared to saline–alkali stress. This strategy of “sacrificing primary root elongation to prioritize lateral root formation” enhances water and nutrient absorption by increasing the root surface area, providing a structural basis for seedling survival. This phenotypic plasticity is a key characteristic of “Qingjian No. 1” in adapting to the composite adversities of the Qinghai-Tibet Plateau, distinguishing it from the “dominant primary root elongation” response mode of pea varieties in plain regions [15,16] and highlighting the stress resistance specificity of local varieties.

Physiological and biochemical analysis indicated that “Qingjian No. 1” copes with oxidative stress by dynamically regulating the balance between the antioxidant enzyme system and membrane lipid peroxidation, exhibiting a “POD-dominated-multi-enzyme synergistic” defense characteristic under combined stress. Under saline–alkali stress, superoxide dismutase (SOD) activity decreased compared to the control, which may be related to Na^+^ binding to the active center of SOD and inhibiting its function [17]. In contrast, peroxidase (POD) showed greater tolerance to ionic stress and was compensatorily activated under combined stress, becoming the core enzyme for H_2_O_2_ scavenging—consistent with the “POD-dominated antioxidant” pattern observed in castor under alkaline stress [18], suggesting a conserved antioxidant enzyme regulation strategy in leguminous plants under high ionic stress. Under combined stress, POD activity was higher than that of the control and significantly higher than that under single stress; meanwhile, malondialdehyde (MDA) content was lower than in the single stress groups, indicating that elevated POD activity directly alleviates oxidative damage by scavenging H_2_O_2_. Although the activities of SOD and catalase (CAT) were lower than the control, they remained higher than under single drought stress, forming an antioxidant network with “POD as the core and SOD-CAT as auxiliary.” This differential regulation of enzyme activity ratios is consistent with the conclusion from studies on castor under alkaline stress that “antioxidant enzyme combinations are dynamically adjusted according to stress intensity” [19], indicating that “Qingjian No. 1” can achieve a balance between energy consumption and oxidative defense through precise regulation of the enzyme system.

Notably, the decreased malondialdehyde (MDA) content under combined stress differs from the common observation in most studies that “MDA content increases under combined stress” (e.g., in Glycyrrhiza uralensis seedlings [20]), which may be attributed to the unique peroxidase (POD)-mediated reactive oxygen species (ROS) scavenging mechanism of “Qingjian No. 1”.

Adaptive regulation of energy metabolism was also observed: under combined stress, the content of citric acid, a key intermediate in the tricarboxylic acid (TCA) cycle, increased by 1.5 times compared to the control, and the abundance of ATP synthesis-related metabolites such as adenosine triphosphate (ATP) was upregulated. This change provides a material basis for stress responses by enhancing energy metabolism, which is consistent with the trend of “enhanced energy metabolism supporting stress adaptation” in rice seedlings under high-CO_2_ treatment [21,22], reflecting a conserved energy-regulation strategy in plants against abiotic stress.

Furthermore, unique differential metabolites exhibit functional differentiation across different stresses: betaine is specifically upregulated under saline–alkali stress, mitigating ion toxicity by stabilizing protein structures [23]; trehalose is uniquely accumulated under drought stress, protecting cell membrane fluidity to cope with osmotic stress [24]; and lysophosphatidylcholine 18:1 is specifically upregulated under combined stress, regulating the expression of downstream stress-responsive genes by activating phospholipid signaling pathways [25]. This pattern of “shared metabolites ensuring basic adaptation and unique metabolites addressing stress specificity” represents an efficient metabolic regulatory mechanism developed by “Qingjian No. 1” through long-term adaptation to the saline–alkali and drought composite adversities of the Qinghai-Tibet Plateau, sharing commonalities with the metabolic adaptation strategies of Poa crymophila under alpine stress [26].

KEGG pathway enrichment analysis revealed that isoflavonoid biosynthesis, nucleotide metabolism, and the phosphatidylinositol signaling system are the core stress-responsive pathways of “Qingjian No. 1”, exhibiting the characteristic of “pathway synergistic activation” under combined stress; the isoflavonoid biosynthesis pathway was significantly enriched under all three stress conditions, with the content of key metabolites such as genistein and daidzein under combined stress higher than that of the control, and as potent antioxidants, isoflavones can scavenge reactive oxygen species (ROS) by providing hydrogen atoms through their benzene ring structure, showing a significant positive correlation with superoxide dismutase (SOD) activity and directly complementing the function of the enzymatic antioxidant system [27]—which is consistent with the mechanism that “isoflavones enhance stress tolerance through antioxidant activity” in soybean under salt stress [28]; additionally, the content of isoflavone-7-O-glucoside was positively correlated with the number of lateral roots, suggesting that it may enhance plant stress resistance by promoting lateral root formation, a regulatory pathway also reported in the salt stress response of roses [28].

The purine and pyrimidine metabolic pathways were significantly enriched under all three stress conditions, with the content of nucleotides such as adenosine and uridine increasing by 1.6 times compared to the control under combined stress; as precursors for RNA synthesis, nucleotides ensure the transcription efficiency of stress-responsive genes including POD and SOD, while adenosine derivatives can act as second messengers to activate the phosphatidylinositol signaling system. Under combined stress, the content of phosphatidylinositol phosphate was higher than that of the control and showed a positive correlation with the vigor index, indicating that this pathway regulates the balance between plant growth and stress resistance through the coupling of “nucleotide signaling-lipid signaling”—a signaling-coupling mechanism that has been reported in soybean responses to salt stress [29].

The phosphatidylinositol signaling system is specifically activated under combined stress; as a key cell membrane component, phosphatidylinositol-4,5-bisphosphate (PIP_2_) can be hydrolyzed to generate inositol trisphosphate (IP_3_) and diacylglycerol (DAG) to activate the calcium signaling pathway while directly regulating ion channel activity to alleviate ion imbalance under combined stress [21]. This specific activation is the core metabolic characteristic that distinguishes the combined stress response of “Qingjian No. 1” from single-stress responses and also serves as a key regulatory node for its adaptation to “osmotic-ion” dual stress, which is consistent with the rule that “the phospholipid signaling pathway dominates ion balance” in tomato under combined stress [27].

Based on the above findings, practical guidance is provided from two aspects: isocorydine and primulasaponin can serve as potential biomarkers for combined saline–alkali and drought stress, enabling rapid screening of superior germplasm via targeted metabolomics [30], while key genes such as chalcone synthase (CHS) in the isoflavonoid biosynthesis pathway and adenosine kinase (ADK) in the nucleotide metabolic pathway can be used as genome editing targets—a strategy successfully applied in the Pisum genus to enhance stress tolerance [31,32]; in terms of cultivation strategy optimization, given the significant increase in peroxidase (POD) activity under combined stress, foliar application of POD activators (e.g., salicylic acid) can enhance the plant’s antioxidant capacity [27], and considering the adaptive significance of lateral root formation to combined stress, “moderate drought pretreatment” (e.g., 10% PEG seed soaking) before sowing can induce root architecture optimization and improve the field emergence rate [33], additionally, combined stress significantly increases isoflavone content—an important nutritional component of forage peas [34]—indicating that cultivating “Qingjian No. 1” in the saline–alkali and arid regions of Qinghai can achieve the synergistic improvement of “stress resistance-nutritional quality”.

This study only focuses on the germination stage (8 days), and the long-term stress response of “Qingjian No. 1” during the tillering stage (30 days) may differ [35]. Additionally, the causal relationship between differential metabolites and stress resistance needs to be verified through gene overexpression or knockout experiments [36]. Furthermore, the symbiotic relationship between leguminous plants and rhizobia is known to significantly enhance abiotic stress tolerance [37], and this study did not address the role of this symbiotic system.

Future research should focus on three aspects: (1) integrate transcriptomic and proteomic data to construct a “gene-protein-metabolite” regulatory network, identifying key regulatory nodes in the isoflavone synthesis and nucleotide metabolic pathways; (2) conduct field validation experiments to evaluate the applicability of biomarkers across different ecological regions and optimize pretreatment cultivation techniques; (3) explore the synergistic stress resistance mechanisms between rhizobia and “Qingjian No. 1”, screen compatible salt-tolerant rhizobial strains, and provide more comprehensive technical support for the efficient cultivation of forage peas in saline–alkali and arid regions.

## 5. Conclusions

This study, set against the unique adverse conditions of the Qaidam Basin on the Qinghai-Tibet Plateau, systematically investigated the response mechanisms of the local superior variety “Qingjian No. 1” forage pea to drought, saline–alkali, and their combined stresses during the germination period by integrating phenotypic observation, physiological and biochemical detection, and non-targeted metabolomics techniques. The main research conclusions are as follows:

1. The combined stress of saline–alkali and drought has a stronger inhibitory effect on the phenotype of “Qingjian No. 1” during the germination period compared to single stress. It adapts to adversity by optimizing its configuration through “sacrificing the elongation of the main root and prioritizing the recovery of lateral roots.”
2. Peroxidase (POD) activity is significantly enhanced, serving as the core enzymatic component for scavenging hydrogen peroxide (H_2_O_2_) and alleviating oxidative stress. It effectively reduces the level of membrane lipid peroxidation, which is specifically reflected as a significant decrease in malondialdehyde (MDA) content, thereby safeguarding the structural and functional integrity of seedling cells during the germination stage.
3. Isoflavone biosynthesis and the phosphatidylinositol signaling system synergistically mediate the response to combined stress, with genistein (2.7-fold increase) and PIP2 (1.8-fold increase) as key metabolites, and isocorydine, primula saponin, and proline as potential stress-resistant biomarkers.

## Data Availability

The original contributions presented in this study are included in the article. Further inquiries can be directed to the corresponding author.

## References

[1] Huang X.Q., Li W.X., Wang J., Li Q., Shen Y., Cheng Y.X., Li T.G., Wang T.L., Wang Y.P., Song L.J. (2024). NaCl stress on physio-biochemical, phenolics synthesis and antioxidant system of pea (*Pisum sativum* L.) sprouts. LWT.

[2] Khan M.A.H., Baset Mia M.A., Quddus M.A., Sarker K.K., Rahman M., Skalicky M., Brestic M., Gaber A., Alsuhaibani A.M., Hossain A. (2022). Salinity-Induced Physiological Changes in Pea (*Pisum sativum* L.): Germination Rate, Biomass Accumulation, Relative Water Content, Seedling Vigor and Salt Tolerance Index. Plants.

[3] Castroluna A., Ruiz O.M., Quiroga A.M., Pedranzani H.E. (2014). Effects of salinity and drought stress on germination, biomass and growth in three varieties of *Medicago sativa* L. Av. Investig. Agropecu..

[4] Yan L., Zhao Y.K., Zhu H., He C., Jin M., Hu F., Liu W. (2023). Seed germination and seedling physiological characteristics of *Zygophyllum xanthoxylum* under salt and drought stress. Pratacult. Sci..

[5] Ma Q.L., Kang J.M., Long R.C., Cui Y.J., Zhang T.J., Xiong J.B., Yang Q.C., Sun Y. (2016). Proteomic analysis of salt and osmotic-drought stress in alfalfa seedlings. J. Integr. Agric..

[6] Mendes G.C., Reis P.A.B., Cali I.P., Carvalho H.H., Aragão F.J.L., Fontes E.P.B. (2013). GmNAC30 and GmNAC81 integrate the endoplasmic reticulum stress- and osmotic stress-induced cell death responses through a vacuolar processing enzyme. Proc. Natl. Acad. Sci. USA.

[7] Ahmad S., Jeridi M., Siddiqui S., Shah A.Z., Ali S. (2023). Genome-wide identification, characterization, and expression analysis of the Chalcone Synthase gene family in *Oryza sativa* under Abiotic Stresses. Plant Stress.

[8] Hamouda M.M., Badr A., Ali S.S., Adham A.M., Sayed Ahmed H.I., Saad-Allah K.M. (2023). Growth, physiological, and molecular responses of three phaeophyte extracts on salt-stressed pea (*Pisum sativum* L.) seedlings. J. Genet. Eng. Biotechnol..

[9] Bibi K., Khan S.A., Ahmad R., Nazir A., Ismail A.M., El-Beltagi H.S., Shalaby T.A., Almaghasla M.I. (2025). Morphological, Biochemical, and Gene Expression Responses of Selected Pea (*Pisum sativum* L.) Varieties to Water-deficit Stress. Rom. Agric. Res..

[10] Devi J., Sagar V., Dubey R.K., Kumar R., Bahadur A., Verma R.K., Rai N., Behera T.K. (2025). Phenotypic, stability and adaptation analysis of vegetable pea (*Pisum sativum* var *hortense* L.) genotypes for high-temperature stress tolerance. Sci. Hortic..

[11] Bao H.J., Zhang H.P., Wang W., Pu X.J., Du M.C., Du W.H., Liu J. (2024). Responses of 14 triticale germplasms to saline-alkali stress at germination stage. Seed.

[12] Chen K., Xu L.J., Yuan Z., Li S.G., Yi J. (2011). Effect of water-soluble cadmium, zinc and their combined pollution on root growth of three kinds of beans. Environ. Chem..

[13] Wang H.Y., Yun P., Shabala L., Chen Z.H., Zhou M.X., Shabala S. (2025). Genotypic variations in sensitivity of root K^+^ and Ca^2+^ transporters to H_2_O_2_ explains differential salt tolerance in wheat and barley. Environ. Exp. Bot..

[14] Li Q., Chen B., Li C.J., Yang Z.Y., Ni R., Chen L.H., Liu N.N., Mao P.Z., Zhang L., Guo X.Y. (2025). Synergistic responses of physiological, transcriptomic, and metabolomic levels in soybean (*Glycine max* (Linn.) Merr) under combined salt-alkali stress. Ind. Crops Prod..

[15] Chen M., Fan J.W., Schneider H.M., Hou L., Tian F.P., Zhao H., Li F.M., Du Y.L. (2025). Tradeoffs between root morphology and carboxylate exudation occur in different alfalfa growth stages and soil depths under water and phosphorus stress. Environ. Exp. Bot..

[16] Singh S.S., Singh N.S., Lamare E., Devi N.R., Devi S.A., Kaguijenliu R., Takum B., Upadhyay K.K., Tripathi S.K. (2025). Drought mitigation in plants through root exudate-mediated rhizosphere interactions: Opportunities for future research. Curr. Plant Biol..

[17] Qu Y.G., Zhao K.F. (2003). Comparison of the stress effects of NaCl and Na_2_CO_3_ on *Suaeda salsa* L. J. Plant Physiol. Mol. Biol..

[18] Cui Z.G., Hao F., Dong X., Zhang Y.Y., Gao Y., Wang Y.L., Yang D.W., Yao B.Y., Lin G.L. (2025). Rhizosphere-to-intracellular adaptation in *Ricinus communis* roots under alkali stress: Pentose phosphate and pyruvate pathways mediate cross-dimensional regulation. Ind. Crops Prod..

[19] Gatti N., Serio G., Gentile C., Bertea C.M., Mannino G. (2024). Impact of a biostimulant enriched in betalain degradation products on ROS signaling, proline accumulation, and phytohormone homeostasis. Curr. Plant Biol..

[20] Zhang Z., Ma R.R., Tao Y.H., Ma H.Z., Jiang X.Y., Wang Z.L., Yang Y.L. (2025). Changes of photosynthetic characteristics, stomatal microstructure and proline metabolism in wheat seedlings under different combined treatments of zinc, iron and copper. Ecotoxicol. Environ. Saf..

[21] Spormann S., Neves J., Pereira C., Soares C., Valente I.M., Rodrigues J.A., Martins V., Kaiser E., Fidalgo F. (2025). Dissecting the physiology of wild tomatoes under abiotic stress: Dynamic photosynthesis and metabolic adaptations to combined drought and salinity. J. Plant Physiol..

[22] Feng D., Ge C.H., Xu W.L., Zhang W.J., Zheng C.L., Tang G.M., Adili Y. (2025). Mechanisms of plant extracts in alleviating drought and saline-alkali stress in plants. Ind. Crops Prod..

[23] Choudhary A., Nepovimova E., Rajput V.D., Malik T., Choudhary M., Bhardwaj N., Peter L., Puri S., Kimta N. (2025). Mechanistic understanding of GABA and trehalose in modulating plant response to drought stress. Plant Stress.

[24] Muhammad N., Dong Q., Luo T., Zhang X., Song M., Wang X., Ma X. (2025). New developments in understanding cotton’s physiological and molecular responses to salt stress. Plant Stress.

[25] Li X.Y., Ma Y.B., Li W.L., Li J.H., Li M.J., Li C.X., Wang Y., Yang Y., Ma X.R. (2024). Ploidy identification and chromosome-level genome assembly of Poa crymophila elucidate high-altitude adaptation. J. Integr. Agric..

[26] Yoon G.A., Park S.M. (2014). Antioxidant action of soy isoflavones on oxidative stress and antioxidant enzyme activities in exercised rats. Nutr. Res. Pract..

[27] Sun X.Y., Cao Y.N., Wang Y.X., Wu S.Q., Beta T., Shi X.C., Wang S.Y., Herrera-Balandrano D.D., Laborda P. (2026). Methyl jasmonate enhances isoflavone biosynthesis and antioxidant activities in *Fusarium sulawense*-infected soybean sprouts. Postharvest Biol. Technol..

[28] Zhu J., Li R.C., Fan S.K., Dong F., Liu Y.Y., Wang C.P. (2025). Genome-wide identification and functional analysis of JAZ gene family in Rosa hybrida reveals the positive role of RhJAZ16 in salt stress tolerance. Ind. Crops Prod..

[29] Zhang B.S., Li M.Y., Song Z., Han J.P., Cheng Z.Q., Chen X.J., Han D.Z., Hu Z.B., Liu C.Y., Yang M.L. (2025). Characterization of the soybean ABF gene family and the key regulatory function of GmABF1 in salt stress response. Int. J. Biol. Macromol..

[30] Docampo R., Huang G.Z. (2021). The IP_3_ receptor and Ca^2+^ signaling in trypanosomes. BBA-Mol. Cell Res..

[31] Ullah I., Ullah I., Zhang H.F., Khan M.R., Mateen A., Pei Y.P., Shakeel A., Bhat A.H., Fu C.L., Chen R.G. (2025). Molecular mechanisms and genomic strategies for enhancing stress resilience in pepper crop. Sci. Hortic..

[32] Diya F., Rahioui I., Vallier A., Benhamou S., Sivignon C., Kfoury L., Rizk F., Da Silva P. (2024). Vicia sativa subsp. sativa native to the Middle East comprises Pea Albumin1 b-like homologs: A promising natural biopesticide. Heliyon.

[33] Rehman S.U., Yang J.W., Zhang J., Zhang L.J., Hao X.H., Song R., Chen S.S., Wang G.P., Hua L. (2025). Salt stress in wheat: A physiological and genetic perspective. Plant Stress.

[34] Liu Z.H., Yang Q., Liu X.B., Li J.P., Zhang L., Chu W., Lin J.C., Liu D.B., Zhao D.Y., Peng X. (2025). Suppression of TaHDA8-mediated lysine deacetylation of TaAREB3 acts as a drought-adaptive mechanism in wheat root development. Mol. Plant.

[35] Kumar S., Pandey G. (2020). Biofortification of pulses and legumes to enhance nutrition. Heliyon.

[36] Cheng X.R., Li J., Chen G.M., Zhou Z.W., Zhu T., Sun Y.P., Dong X.O., Liu L., Chi W.C., Dai Z.Y. (2025). A BAHD acyltransferase STBR1 confers both saline-alkali tolerance and blast resistance by stabilizing the non-canonical catalase CATA to promote H_2_O_2_ scavenging in rice. Plant Commun..

[37] Mir M.I., Kumar B.K., Gopalakrishnan S., Vadlamudi S., Hameeda B. (2021). Characterization of rhizobia isolated from leguminous plants and their impact on the growth of ICCV 2 variety of chickpea (*Cicer arietinum* L.). Heliyon.

